# “Positive biology”: the centenarian lesson

**DOI:** 10.1186/1742-4933-9-5

**Published:** 2012-04-23

**Authors:** Calogero Caruso, Giuseppe Passarino, Annibale Puca, Giovanni Scapagnini

**Affiliations:** 1 Department of Pathobiology and Medical and Forensic Biotechnologies, University of Palermo, Corso Tukory 211, 90134, Palermo, Italy; 2 Department of Cell Biology, University of Calabria, Rende, Italy; 3 IRCCS Multimedica, Milan, Italy and University of Salerno, Baronissi, Italy; 4 Department of Health Sciences, University of Molise, Campobasso, Italy

**Keywords:** Ageing, Frailty, Longevity, “Positive Biology”

## Abstract

The extraordinary increase of the elderly in developed countries underscore the importance of studies on ageing and longevity and the need for the prompt spread of knowledge about ageing in order to satisfactorily decrease the medical, economic and social problems associated to advancing years, because of the increased number of individuals not autonomous and affected by invalidating pathologies.

Centenarians are equipped to reach the extreme limits of human life span and, most importantly, to show relatively good health, being able to perform their routine daily life and to escape fatal age-related diseases. Thus, they are the best example of extreme longevity, representing selected people in which the appearance of major age-related diseases, such as cancer, and cardiovascular diseases among others, has been consistently delayed or escaped. To discuss the relevance of genetics and life style in the attainment of longevity, five papers mostly focused on Italian centenarians have been assembled in this series. The aim is to realize, through a” positive biology” approach (rather than making diseases the central focus of research, “positive biology” seeks to understand the causes of positive phenotypes, trying to explain the biological mechanisms of health and well-being) how to prevent and/or reduce elderly frailty and disability.

## Introduction

During the last century, life expectancy at birth rose by a remarkable 30 years in Western countries and in Japan, initially because of reductions in infant, child, and maternal mortality and then because of declining mortality in middle and old age. So, during the past century, humans have gained more years of average life expectancy than in the last 10,000 years: we are now living in a rapidly ageing world. The sharp rise in life expectancy, coupled to a steady decline in birth rates in all developed countries, has led to an unprecedented demographic revolution characterized by an explosive growth in the number and proportion of older people. In 1900, about 40% of babies born in Western countries were expected to live beyond age 65. Today in these same countries more than 88% of all newborns will live past age 65 and at least 44% will live beyond age 85. The number of people aged 60 years or older exceeded 635 million in 2002, and is expected to grow to nearly 2 billion by 2050. The proportion of people aged 60 and over stands about 1 in 4 in many Western countries as well as in Japan. Should the present trend continues, this ratio is expected to reach 1 in 3 by 2050. So, many countries have rising ageing populations and are facing an increased prevalence of age-related diseases and increasing healthcare costs, since the rapid rise in older people is accompanied by an increase in the number of people with chronic age-related diseases. However, the improvement in public health has reduced the principal causes of mortality in the elderly, allowing an increasing number of individuals to reach the maximum lifespan age. Indeed, around the 1950s, in all industrialized countries, the progressive decline of mortality in oldest old people has increased, so that the number of centenarians has augmented about 20 times [1-6]. Nowadays it is reasonable to assume that the total number of centenarians is more than three hundred thousand people worldwide [7].

The increased ability to reach 100 years in industrialized countries over the last 150 years most likely reflects a rise in life expectancy as a consequence of improvements in diet and a reduced exposure to infection and inflammation [1]. In favour of diet as a modulator of longevity, the Elderly Prospective Cohort Study identified a reduced overall mortality among the elderly consuming a modified Mediterranean diet in which saturated fatty acids were substituted for monounsaturated ones [8] and the zones of Sardinia and Sicily characterized by male longevity are characterized by close adherence to Mediterranean diet [9]. The traditional Mediterranean diet provides about 40% of calories from fat, mostly monounsaturated and polyunsaturated fat [8]. Concerning Japanese centenarians, traditional Okinawan diets provide about 90% of calories from vegetables carbohydrate, therefore it is low in calories but nutritionally dense, particularly with regard to vitamins, minerals, and phytonutrients [10]. Concerning inflammation, the reduction in lifetime exposure to infectious diseases and other sources of inflammation, the cohort mechanism, has been suggested to contribute to the historical decline in old-age mortality, suggesting long-life pathogen burden as the most important factor for age-related inflammation. Accordingly, some studies have linked an individual exposure to past infection to levels of chronic inflammation and to increased risk of heart attack, stroke, and cancer [11-14].

The extraordinary increase of the elderly in developed countries underscore the importance of studies on ageing and longevity and the need for the prompt spread of knowledge about ageing in order to satisfactorily decrease the medical, economic and social problems associated to advancing years for the increase of the subjects which are not autonomous and are affected by invalidating pathologies [15].

Recently, it has been pointed out that most biomedical research should be termed ‘negative biology’, because the study of disease is its central heart, reflecting the prevalence of pathology-oriented negative biology, so focusing on the causes of pathology. By contrast, the Author invites to focus on a different approach, named “positive biology”. Rather than making diseases the central focus of research, positive biology seeks to understand the causes of positive phenotypes, trying to explain the biological mechanisms of health and well-being [16]. In particular, concerning our topic, this means to understand why some individuals, i.e. the centenarians, have escaped neonatal mortality, pre-antibiotic era diseases, and fatal outcomes of age-related diseases, so leaving more than 100 years [17,18]. Investigating the biological mechanisms underlying centenarian longevity, therefore, shows an interesting conundrum for positive biology. The knowledge grew out of this approach could allow to modulate the rate of ageing providing valuable information on the lifestyle to achieve healthy ageing. In addition, studying centenarians might supply important indications how to build up drugs that can slow or delay ageing, with benefits for those who are more vulnerable to disease and disability [19,20].

The model of centenarians is not simply an additional model with respect to well-studied organisms, since the study of humans has revealed characteristics of ageing and longevity as geographical and sex differences, role of antigenic load and inflammation, which did not emerge from studies in laboratory model systems and organisms. So, scientists have focused their attention on centenarians as optimal model to address the biological mechanisms of successful ageing [17,18].

Centenarians are equipped to reach the extreme limits of human life span and, most importantly, to show relatively good health, being able to perform their routine daily life and to escape fatal age-related diseases. Thus, they are the best example of extreme longevity, representing selected peope in which the appearance of major age-related diseases, such as cancer, and cardiovascular diseases among others, has been consistently delayed or escaped [17,18].

The ageing process and longevity are multi-factorial events. Genetic, epigenetic, stochastic and environmental factors seem to have a crucial role in ageing and longevity. As well known, life expectancy is a familial trait and longevity is determined by different factors. Epidemiological evidence for a genetic component to variation in human lifespan comes from twin studies and family studies. By comparing life span in twins, researchers have found that approximately 25% of the overall variation in human lifespan can be attributed to genetic factors, which become more relevant for extreme longevity [21-24]. Conditioning factors, that arise in the first part of life (socio-economic state of parents, education and month of birth, which has been found to reflect the environmental conditions during the prenatal and early postnatal period), account for another 25% of such variability; life circumstances at adult and old age (including socio-economic status and medical assistance) may account for about the remaining 50% [25]. In this context, the study of centenarian offspring, a group of healthy elderly people with a familiar history of longevity, might help gerontologists to better identify the correlation between genetic profile and hope of a healthy ageing. Previous studies have reported that centenarian offspring, like their centenarian parents, have genetic and immune system advantages, which reflect a minor risk to develop major age-related diseases, such as cardiovascular diseases, hypertension or diabetes mellitus as well as cancer [26,27].

### The series

To discuss the relevance of genetics and life style in the attainment of longevity, five papers mostly focused on Italian centenarians have been assembled in this series with aim to understand, through a “positive biology” approach, how to prevent and/or reduce elderly frailty and disability.

As it is known, healthy ageing and longevity in humans result from a number of factors, including genetic background, favorable environmental and social factors and chance. So, in their paper Montesanto et al., discuss the role of epidemiological, genetic and epigenetic factors in the variation of quality of ageing and lifespan, including the most promising candidate genes investigated so far. Epigenetic modifications indicate the sum of heritable changes, such as DNA methylation, histone modification and miRNA expression, that affect gene expression without changing the DNA sequence. So, they outlined some recent advance in the epigenetic studies of ageing, as epigenetics, a bridge between genetics and environment, might explain many aspects of ageing and longevity.

In their review, Ferrario et al,. point out that the genetic origin of exceptional longevity and the more recently observed environment-driven increase in the average age of the population could possibly be explained by the same genetic variants and environmentally modulated mechanisms. The potential overlap of hits for environmentally and genetically mediated predisposition for extreme longevity in centenarians is highlighted by the association of genetic variants of genes that regulate, or that are regulated by, nutrient metabolism. They conclude that the adoption of innovative study designs combined with novel genetic platforms and innovative statistical methods hopefully will bring to the identification of new intervention points at which to modulate ageing and the diseases of ageing.

In their review, Balistreri et al., report their data gathered for over 10 years in Sicilian centenarians. Based on their findings, they suggest longevity as the result of an optimal performance of immune system and an over-expression of anti-inflammatory sequence variants of immune/inflammatory genes. The data from Sicilian investigation add another piece to complex puzzle of genetic and environmental factors involved in the control of life span expectancy in humans, showing a complex network of trans-inactive genes able to influence the type and strength of immune responses to environmental stressors, and as final result, conditioning individual life expectancy.

The paper of Vasto et al., pays attention on the modifiable lifestyle factors such as diet and nutrition to achieve extension of health span. Previous data reported that in Sicily, the biggest Mediterranean island, there are some mountain regions where there is a high frequency of male centenarians with respect to the Italian average. The present data show that in Sicani Mountain zone there are more centenarians with respect to the Italian average. In fact, in five villages of Sicani Mountains, there where 19 people with age ranging from 100–107 years old, on the total population of 18,328 inhabitants. So, the centenarian number was more than 4.32-fold higher the national average (10.37 vs. 2.4/10,000); the female/male ratio was 1.1:1 in the study area, while the national ratio is 4.54:1. Unequivocally, their nutritional assessment showed a high adherence to the Mediterranean nutritional profile with low glycemic index food consumed.

In their paper, Davinelli et al., focus on dietary patterns of centenarians and nutrient-sensing pathways that have a pivotal role in the regulation of life span. They point out that the realization of healthy longevity is possible but to achieve a longer and a healthier life, increased attention must be placed on lifestyle choices, particularly the diet. To date the main dietary intervention that may retard the ageing process is calorie restriction and a typical example is the Okinawan population in Japan. Many of the genes that act as key regulators of lifespan also have known functions in nutrient sensing, thus called nutrient-sensing longevity genes and variant associated to longevity have been described.

## Conclusion

Success in increasing longevity in laboratory organisms has shown that ageing is not an immutable process [28]. Hence, the time has come to get more serious about the effort to slow human ageing or to age successfully. On the other hand, if ageing is combined with extended years of healthy life, it could also produce unprecedented social, economic, and health dividends [29,30]. Thus, particular attention has been centered on centenarian genetic background, immune system and life style.

Centenarians, despite being exposed to the same environmental conditions as members of the average population, manage to live much longer; moreover, as a consequence of demographic selection, centenarians have a compression of morbidity and mortality towards the end of their life-span [31]. On the other hand, it seems that long lived individuals harbor genetic risk factors for age-related diseases as recently underlined also by genome-wide association studies data, reporting as very long lived individuals share the same number of risk alleles for coronary artery disease, cancer, and type 2 diabetes than younger controls from the same population, thus suggesting that human longevity is not compromised by the cumulative effect of a set of risk alleles for common disease [32-34]. These studies support the existence of buffering mechanisms operating in the determination of human longevity, probably through the presence of favorable genotypes contrasting the deleterious effect of age-related disease genes: as a result, the frequency of deleterious genotypes may increase among individuals with extreme lifespan because their protective genotype allows disease-related genes to accumulate [35]. A better understanding of the functional genes that affect healthy longevity in humans may lead to a rational basis for intervention strategies that can delay or prevent age-related diseases. However, with the exception of APOE and FOXO3A variants, none of the many candidate genetic variants tested to date have been consistently replicated across populations. This is possibly on account of differing environmental stimuli generating inconsistent demographic pressures, making results, as a consequence, irreproducible [36].

Regarding immunological aspects, studying centenarian offspring reveal that several B cell immune parameters are better preserved than in age-matched controls, and together with their genetic background could contribute to their healthier ageing. This suggests the idea of the “familiar youth” of the immune system [37,38].

Concerning life style, it is out of doubt that healthy centenarians live surrounded by a solid support network of friends and family. However, diet plays a key role in successful ageing. Specific dietary factors that may be involved include a high intake of fruit and vegetables, and in the property of phytochemicals to activate specific nutrient sensing pathways. Centenarians have a very high intake of phytochemicals in the diet. All plants contain these natural compounds and the elderly have significantly lower levels of lipid peroxidation and they suffer less free-radical-induced damage [39-41]. From a scientific perspective, a particular diet able to delay ageing may help to identify new molecules to extend and ameliorate lifespan, opening new opportunities for drug discovery and companies working in nutrition and pharmacology. As recently stated [16], drugs able to mime the effects of calorie restriction might postpone most age-related diseases. In this manner, we could get a much greater health bonus for elderly than overcoming any one definite age-related disease. It has been calculated, in fact, that the slowdown of ageing rate by seven years might reduce the age-specific risk of death and frailty by about half at every age [16,30].

In conclusion, the development of strategies that will lead to the extension of healthy life and that would result in slowing the rate of ageing may be part of the new paradigm for the medical sciences that is the ‘positive biology’.

## Competing interests

The authors declare that they have no competing interests.

## Authors’ contributions

CC wrote the paper. All authors edited the paper and approved its final version.
